# Percussion Drilling of Deep Holes Using Picosecond Ultrashort Pulse Laser in Ni-Based Superalloy Coated with Ceramic Thermal Barrier Coatings

**DOI:** 10.3390/ma13163570

**Published:** 2020-08-13

**Authors:** Haodong Liu, Wanqin Zhao, Lingzhi Wang, Xiaowei Shen, Ning Wang, Xu Wang

**Affiliations:** 1 School of Materials Engineering, Shanghai University of Engineering Science, Shanghai 201620, China; M050119308@sues.edu.cn (H.L.); 18321261951@163.com (L.W.); M050119311@sues.edu.cn (X.S.); 17805052071@163.com (N.W.); wangxu18895373675@163.com (X.W.); 2 State Key Laboratory for Manufacturing Systems Engineering, Xi’an Jiaotong University, Xi’an 710049, China; 3 Shanghai Collaborative Innovation Center of Laser Advanced Manufacturing Technology, Shanghai 201620, China

**Keywords:** percussion drilling, ablation, deep hole, Ni-based superalloy, ceramic TBCs

## Abstract

Ni-based superalloy with ceramic thermal barrier coatings (TBCs) is a composite material, which can be used in special environments with high temperature and high pressure such as aeroengine blade. In order to improve the cooling effect of the aeroengine, it is necessary to perform multi-size and large-area holes processing on the surface of blades. As a non-contact processing method with fast processing speed, good processing quality and almost no deformation, laser processing has been one of the important processing methods for film cooling hole processing of aeroengine blades. Percussion drilling is presented using picosecond ultrashort pulse laser in order to explore processing of deep holes in Ni-based superalloy, ceramic TBCs, and ceramic TBCs/substrate multilayer material. The effects of pulses, threshold and wavelength on hole diameter have been discussed, and the experiment on the deep hole ablation with 1064 nm wavelength has been performed. By analyzing the hole size and morphological characteristics of multiple processing parameters, the variation of hole cylindricity is obtained. A high-quality hole, without spatters around the periphery of hole entrance and without recast layer on the side-wall surface, in Ni-based superalloy coated with ceramic TBCs has been drilled. This research has potential applications to blade film cooling holes.

## 1. Introduction

Ceramic thermal barrier coatings (TBCs) have been prepared on metals substrates surfaces to improve the properties of materials, such as the high temperature properties, abrasion resistance and corrosion resistance [1]. There are many applications for ceramic TBCs/substrate multilayer material. One of them is fabrication of the turbine blades for aircraft engines, using Ni-based superalloy coated with ceramic TBCs [2,3]. There are good material stability, good strength at high temperatures, and good corrosion and oxidation resistance for the Ni-based superalloy. However, preparing ceramic TBCs on the surface of blades made of Ni-based superalloy and drilling many cooling holes [4,5] are also needed to adapt to working environment of turbines’ blades, which see very high temperature durability in such harsh working environments [6,7]. In a word, the turbine blades are composed of ceramic TBCs/substrate multilayer composite materials. Furthermore, both the ceramic TBCs and Ni-based superalloy are difficult materials to work such as for drilling holes, due to their high hardness. Therefore, traditional machining methods are not practical.

Laser drilling which has its comparable advantages is an outstanding method for material processing [8,9,10]. For example, it is a non-contact machining method that does not cause mechanical deformation of the sample. Without a cutting tool, it does not cause wear or transfer cutting force to the sample. In addition, laser drilling offers high precision, flexibility, and good compatibility with automation. As a result, it is very suitable for complex work [11]. There are several main types of lasers which can be categorized based on their pulse duration, including long, short and ultrashort pulse lasers. For drilling holes, it can be classified according to the relative movement between the laser and the sample. The processing methods with relative movement are trepanning and helical drilling, otherwise it is percussion drilling [12]. However, the potential downsides of laser drilling are mainly the need to contain the molten spatters around the periphery of holes and the recast layer on the side-wall surface. For example, Sun et al. investigated that millisecond long pulse laser trepanning holes in Ni-based superalloy coated with ceramic TBCs. It was found that there were a large number of molten spatters on the holes surfaces and the recast layer up to the micron scale, interfacial cracks and cracks extension on the holes side-wall surfaces, which affected severely the mechanical strength characteristics [13,14]. Wang et al. found that the crack width at BC (bond coat)/substrate dropped from 8 to 15 μm in millisecond laser processing [15]. Qi et al. researched the ablation of metals coated with ceramic TBCs using nanosecond pulse fiber lasers, and found that the recast layer, edge protrusions and microcracks could not be eliminated due to the thermal effects during nanosecond laser drilling [16]. Fan et al. used Nd: YAG (JK300D Nd: YAG pulsed laser, GSI, Birmingham, UK) pulsed laser to process Ni-based superalloy with TBCs, and found the maximum crack length reaches up to approximate 320 μm and the maximum crack width is approximate 50 μm [17]. In picosecond and femtosecond ultrashort pulse laser machining, these problems can be significantly improved, but for hole drilling [18,19,20], there is still a recast layer which thickness is about 5 µm after femtosecond ultrashort pulse trepanning [20]. Comparing to trepanning and helical drilling, percussion drilling offers high efficiency due to the lack of relative movement between the laser and the work. Feng et al. compared percussion and trepanning drilling for single-crystal superalloy coated with ceramic TBCs using a femtosecond laser. Their research showed that it took 10 minutes to drill through a sample with a thickness of 0.8 mm using trepanning drilling, and only 5 minutes and 3 minutes, respectively, for work samples with thicknesses of 1.2 mm and 0.6 mm using percussion drilling [21].

Overall, compared with the nanosecond and millisecond pulses lasers, the ultrashort pulse laser has obvious advantages in high quality hole processing. Meanwhile, the processing efficiency of percussion drilling is higher than the one of trepanning/helical drilling. In this paper, percussion drilling of deep holes in ceramic TBCs, Ni-based superalloy coated without and with ceramic TBCs using picosecond ultrashort pulse laser is presented. First, the effects of threshold and wavelength on hole diameter have been discussed in order to study the hole ablating with the diameter larger than 100 µm, which is the value required by blade film cooling hole. Then, the experiment on the deep holes ablation with 1064 nm wavelength has been performed. The hole dimension and morphological characteristics have been analyzed for multiple processing parameters. At last, a high-quality hole, with no spatters around the periphery of hole entrance, and without recast layer on the side-wall surface, in Ni-based superalloy coated with ceramic TBCs, has been drilled. This research has potential applications to blade film cooling holes.

## 2. Experiments and Samples

### 2.1. Experimental Setups and Parameters

**Figure 1:**
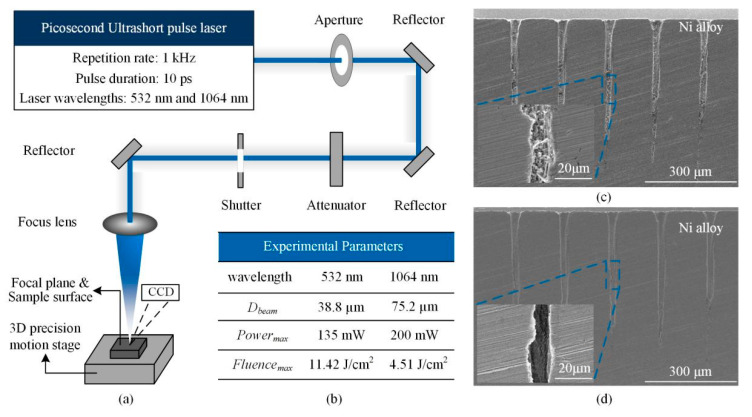
Machining system, experimental parameters and SEM images of hole profile (**a**): Schematic of the picosecond ultrashort laser micro-machining system; (**b**): Experimental Parameters; (**c**): SEM images of the hole profile before cleaned; (**d**): SEM images of the hole profile after cleaned.

Figure 1a illustrates the schematic of the picosecond ultrashort pulse laser micro-machining system. The 10 ps laser used for irradiation in the experiments was a neodymium-vanadate (IC-1500 ps Nd:VAN REG AMP, High Q Laser, Rankweil, Austria ) laser with wavelengths of 532 nm and 1064 nm and the repetition rate of 1 kHz. The focal plane of the laser beam was placed on the sample surface using the optical lens with the focus length of 200 mm. After the beam passed through the aperture, the focused beams diameters were 38.8 and 75.2 µm at 532 and 1064 nm wavelengths, respectively. This moment, as shown in Figure 1b, the maximum powers and fluences were 135 mW and 11.42 J/cm^2^, 200 mW and 4.51 J/cm^2^ at 532 and 1064 nm wavelengths, respectively. Besides, when there was no aperture on the transport path of 1064 nm wavelength, the related parameters were 36.1 µm, 248 mW and 6.06 J/cm^2^. All these are the experimental parameters in this paper.

The experiments were performed under ambient conditions. After the experiments, the hole diameter and surface morphology were measured using scanning electron microscopy (SEM), the hole diameter was the average value of the transverse and longitudinal diameters. Then, the holes were split and cleaned with acetone in an ultrasonic cleaner (SEM images of holes before and after cleaned could be found in Figure 1c,d, demonstrating the importance of cleaning). The hole side-wall morphology was subsequently measured from the SEM micrographs. The samples were then polished using standard metallographic procedures, the polished surfaces were etched with a specialized solution used with the Ni-based superalloy to reveal micro-structural features.

### 2.2. Samples

**Figure 2:**
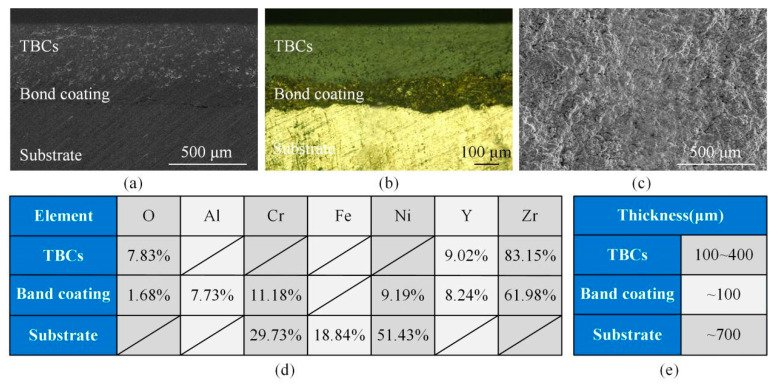
Ceramic thermal barrier coatings (TBCs)/substrate multilayer materials (**a**): SEM image (**b**): Metallographic image (**c**): Surface of TBCs (**d**): Element contents of multilayer materials (**e**): Thickness of the multilayer materials.

One of the experimental samples was the Ni-based superalloy coated with ceramic TBCs, shown in Figure 2. The ceramic TBCs/substrate multilayer materials were composed of the ceramic TBCs, the bonded coating with similar properties to the metal substrate, and the substrate Ni-based superalloy, as shown in Figure 2a,b. The surface morphology of sample is shown in Figure 2c. Figure 2d shows that the elements. -Element content of layers were different and the elements with the most contents of ceramic TBCs, band coating and substrate are Zr, Zr and Ni, respectively. The thicknesses of the ceramic TBCs, the bonded coating, and the substrate shown in the Figure 2e were approximately 0.1~0.4 mm, 0.1 mm, and several millimeters, respectively. Another experimental sample was the Ni-based superalloy with a thickness of 1 mm and the ceramic TBCs with a thickness of 0.7 mm.

## 3. Results and Discussions

### 3.1. Hole Diameter Versus Pulses and Threshold of Material with 532 nm Wavelength

**Figure 3:**
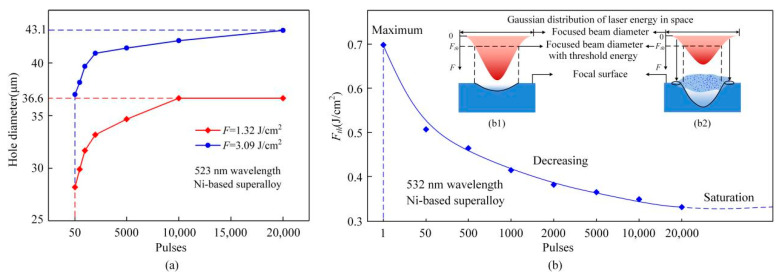
Hole diameter and threshold versus pulses (**a**): Hole diameter versus pulses (**b**): Threshold versus pulses (**b1**): Idealistic relationship between bean diameter and hole diameter with single pulse ablation (**b2**): Influencing factors of the hole diameter with pulse laser ablation.

Figure 3 shows the relationship of hole diameters and thresholds with pulses from 50 to 20,000. With the increase of pulses, it can be confirmed that the holes diameters increased gradually, as shown in Figure 3a. However, the tendency of increase gradually slowed down until saturation, a hole with maximum hole diameter appeared. For example, there would be a largest hole with a diameter of 36.6 μm when the fluence was 1.32 J/cm^2^. The thresholds of Ni-based superalloy and the single-pulse threshold have been calculated based on the fracture diameter method and the empirical incubation model, respectively (The detailed computation mothed can be found in references of [22,23]). The results have been shown in Figure 3b and it could be found that the threshold decreased gradually with the increase of pulses. Furthermore, it has been believed that the threshold would reach to the saturation state with the increase of pulses ultimately on account of the previous researches [24,25]. Overall, the highest threshold existed for the single pulse ablation, and the hole diameter, theoretically, was equal to the cross-sectional diameter of focused beam at the threshold fluence in the case of single-pulse ablation as shown in Figure 3b1. Thereafter, with pulses increasing, the threshold decreased but the hole diameter increased gradually. Finally, the hole diameter reached the saturation state when the threshold was saturated. However, there were many influencing factors on hole diameter with pulse laser ablation as shown in Figure 3b2, mainly including the effect of plasma clouds, the cumulative effect of the surrounding fluence lower than the threshold fluence except the threshold of material. Firstly, the atmospheric and material plasma cloud being in the inlet would further expand the hole diameter. Moreover, the laser beam was scattered by the plasma cloud making the hole diameter expanding. In addition, the fluence of continuous accumulation at the edge below the material threshold would lead to the ablation, the hole diameter became large.

**Figure 4:**
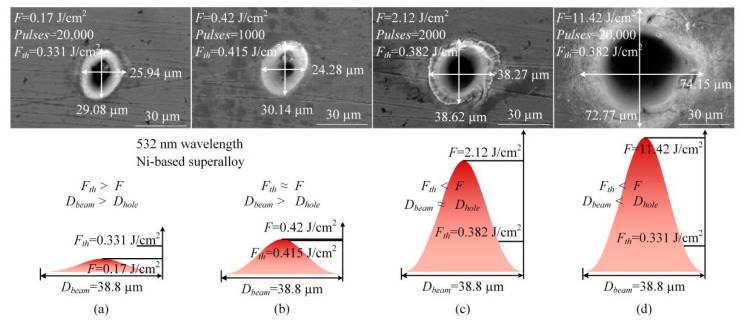
Hole diameter and beam diameter with different relationship of size between thresholds and fluences (**a**): Threshold > Fluence (**b**): Threshold ≈ Fluence (**c**): Threshold < Fluence (**d**): Threshold < Fluence.

Figure 4 illustrates the SEM images of holes with different relationship of size between thresholds and fluences. Although the laser fluence of 0.17 J/cm^2^ was smaller than the threshold of 0.331/cm^2^, as shown in Figure 4a, a hole still could be drilled with a certain pulses, and the hole diameter was smaller than the beam diameter of 38.8 µm. When the fluence was almost equal to the threshold, as shown in Figure 4b, the hole diameter was still smaller than the beam diameter. Continuing to increase the experimental parameters, we could obtain that the hole diameter was about same as the beam diameter as shown in Figure 4c. After that, the fluence continued to increase to the maximum of 11.42 J/cm^2^ at the wavelength of 532 nm; the hole diameter was 73.46 µm.

**Figure 5:**
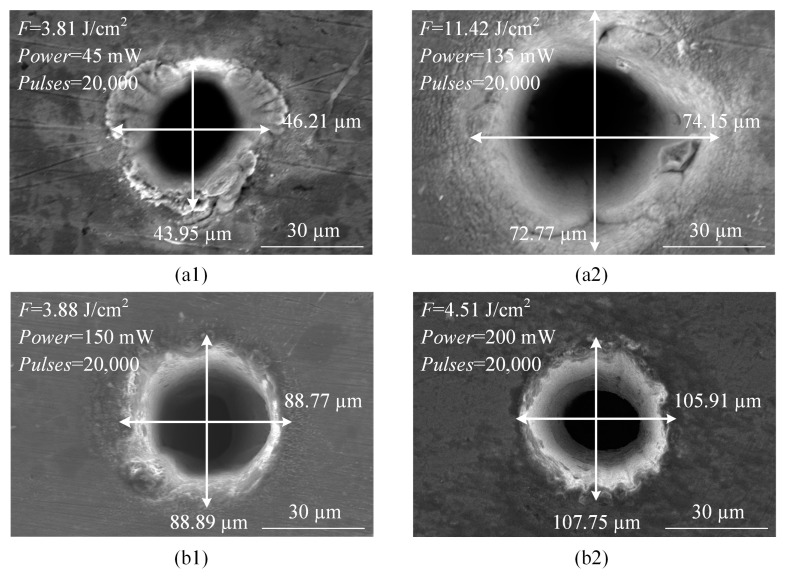
Hole diameter versus laser wavelength (**a1**,**a2**): 532 nm wavelength, *D_beam_* = 38.8 µm (**b1**,**b2**): 1064 nm wavelength, *D_beam_* = 75.2 µm.

Compared with the ablation at 532 nm wavelength, the holes diameters ablated at 1064 nm were obviously large. Figure 5 shows the SEM images of holes at two wavelengths, the beam diameters were 38.8 and 75.2 µm at 532 and 1064 nm wavelengths, respectively. When the laser fluences were almost the same as shown in Figure 5a1,b1, the hole diameter at 1064 nm wavelength was approximately twice that at 532 nm wavelength. When the fluences were at a maximum of 11.42 J/cm^2^ at 532 nm wavelength and 4.51 J/cm^2^ at 1064 nm wavelength, the corresponding ablated holes diameters, shown in Figure 5a2,b2, were 73.46 and 106.83 µm, respectively. In other words, although the fluence at 1064 nm wavelength is low, the hole diameter is obviously larger than that at 532 nm wavelength due to its larger beam diameter.

### 3.2. Deep Holes Ablation with 1064 nm Wavelength

**Figure 6:**
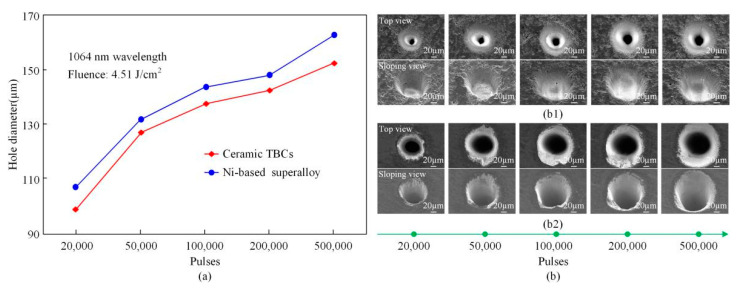
Holes diameters and SEM images in ceramic TBCs and Ni-based superalloy (**a**): Hole diameter (**b**): SEM image of holes (**b1**): Ceramic TBCs (**b2**): Ni-based superalloy.

The above research shows that the hole with large diameter could be obtained for ultrashort pulse laser drilling with 1064 nm wavelength. Then, the comparative study on deep hole ablation in ceramic TBCs and Ni-based superalloy by using picosecond ultrashort pulse laser with 1064 nm wavelength would be carried out. For the hole diameter, as shown in Figure 6a, the holes diameters increased gradually with the increase in the pulses for both materials, and the holes diameters in Ni-based superalloy were larger than those in ceramic TBCs. Moreover, the SEM images of the holes surface morphologies in ceramic TBCs and the Ni-based superalloy are shown in Figure 6b. It could be seen that there were no spatters around the periphery of the holes entrances in the ceramic TBCs, shown in Figure 6b1. The side-wall morphology near the holes entrances was made up of fine ripples. There were some small voids on the surfaces of the side-walls, which were not induced by the laser ablation, but derived from the process of preparing ceramic TBCs. In addition, there were small amounts of molten spatters around the periphery of the hole entrances in the Ni-based superalloy. The side-wall morphology was also composed of fine ripples with some stripes on the side-wall surface, as shown in Figure 6b2. Furthermore, it was found that the holes surface morphological quality did not deteriorate even for more pulses. The spatters were gone with increased pulses, for the holes ablated in Ni-based superalloy as shown in Figure 6b2. The reason of the phenomenon is the self-cleaning effect, and its effectiveness was presented in reference of [26].

**Figure 7:**
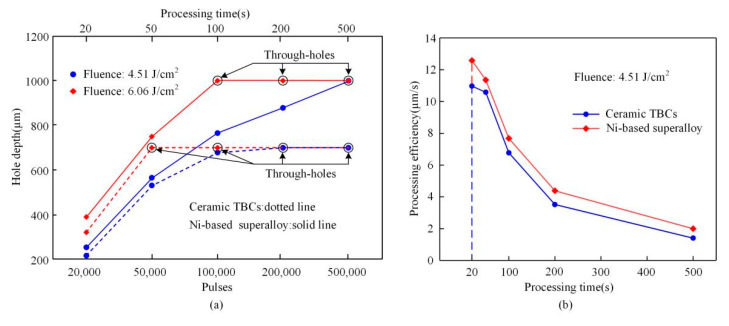
Holes depths and processing efficiency in ceramic TBCs and the Ni-based superalloy (**a**): Hole depth versus pulses (**b**): Processing efficiency versus time.

Figure 7 illustrates the holes depths and processing efficiency versus processing time in ceramic TBCs and the Ni-based superalloy. Holes depths shown in Figure 7a, similar with the holes diameters, increased gradually with the increase in the pulses for both materials, and the holes depths in Ni-based superalloy were deeper than that in ceramic TBCs. When the fluence was 4.51 J/cm^2^, after 500,000 pulses, the Ni-based superalloy sample was penetrated by an ablated through-hole. With a repetition rate 1 kHz, the processing time of a through-hole with the thickness of 1 mm in the Ni-based superalloy was 500 s. For the fluence of 6.06 J/cm^2^, after 100,000 pulses, the through-hole completed, meanwhile, the processing time was only 100 s. On the other hand, for ablation of the TBCs with a thickness of 0.7 mm, the through-hole was drilled first with a fluence of 6.06 J/cm^2^, 50,000 pulses, and a processing time of 50 s. When the laser fluence was 4.51 J/cm^2^, the processing time for a through-hole was 200 s. Overall, for the ablation of both Ni-based superalloy and ceramic TBCs, the processing efficiency was much higher for the high laser fluence. In addition, Figure 7b takes the fluence of 4.51 J/cm^2^ as an example to compare the processing efficiency of Ni-based superalloy and ceramic TBCs, that is, the removal depth per unit time. It can be seen from the Figure 7b that the processing efficiency of Ni-based superalloy was greater than that of ceramic TBCs, and the highest processing efficiency was at the start of 20 s. As the laser processing progressed, the processing efficiency gradually decreased until the processing ended. In other words, the ablation depth of each pulse in laser pulse processing was different.

**Figure 8:**
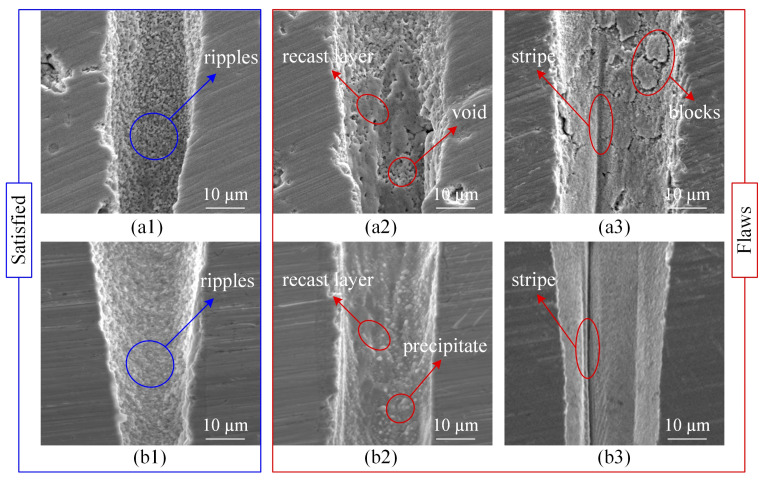
SEM images of typical morphological features on the holes side-walls surfaces. (**a1**–**a3**): Ceramic TBCs (**b1**–**b3**): Ni-based superalloy.

For the side-wall morphology, there were many similar characteristics even for different metal Ni-based superalloy and ceramic TBCs. These morphologies as shown in Figure 8 came from part of the holes under different processing parameters. The ripples shown in Figure 8a1,b1 represent a satisfactory morphology, while, the recast layers, blocks and stripes belong to the flaws shown in Figure 8a2,a3,b2,b3. Based on the thermal effect during the interaction between laser and material, the materials melt and recast on the surface of hole side-wall, it is named the recast layer [27]. Therefore, the joint strength between recast layers and substrate has been weaken. Similar with the recast layer, there is also the weaken joint strength between blocks and substrate. As for stripes, the further studying needs to be taken to investigate their cause. Certainly, there are some unique flaws for each material, such as the voids and blocks on the side-wall surface of TBCs and the precipitates on the side-wall surface of Ni-based superalloy as shown in Figure 8a2,a3,b2, respectively. The cause of the voids may be composed of two parts, one is the defects of ceramic coating [28], and the other is the self-focusing filamentation phenomena in the laser processing [29]. These precipitates are nano-scale and are crystallized by randomly extracting surrounding elements [30]. In particular, flaws are destructive morphologies and lead to poor material performance, which should be reduced or even eliminated as much as possible.

**Figure 9:**
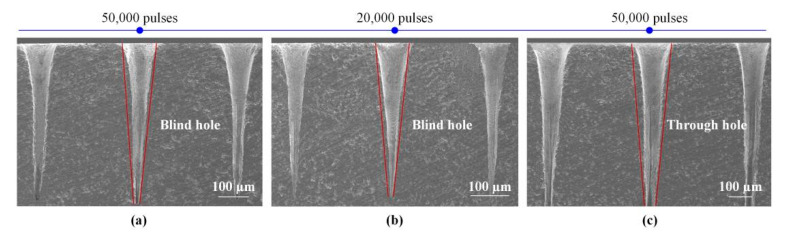
SEM images of holes side-walls in ceramic TBCs. (**a**): Fluence = 4.51 J/cm^2^ (**b**): Fluence = 6.06 J/cm^2^ (**c**): Fluence = 6.06 J/cm^2^.

Fortunately, flaws on the side-wall surface can be eliminated through the regulation of the processing technology. Holes with high quality side-walls could be drilled using percussion drilling in TBCs and Ni-based superalloy, based on picosecond ultrashort pulse lasers. For the holes in TBCs, as shown in Figure 9, there were no recast layers for both blind- and through-holes. In addition, they demonstrated a very high repeatability for the ablated holes using ultrashort laser pulses, which is critical for many industrial applications. However, the tapers of blind- and through-holes, especially the blind holes shown in Figure 9a,b, were excessive. It is a serious issue and a difficulty for the holes ablated using the percussion drilling with ultrashort pulse laser. 

**Figure 10:**
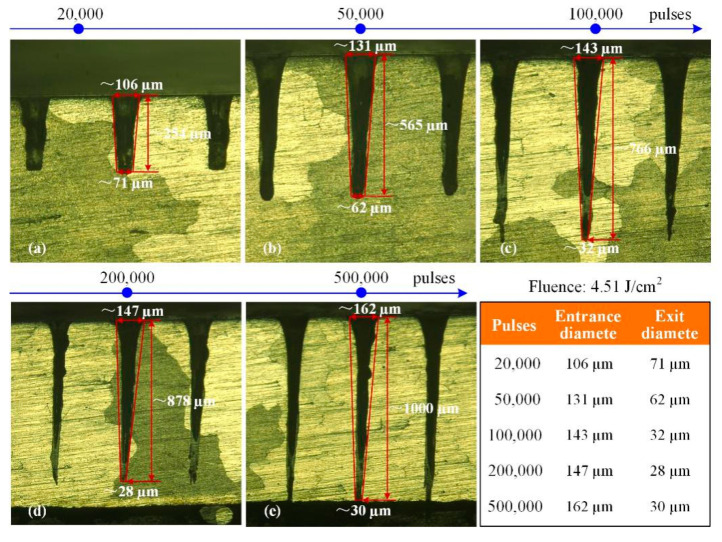
Metallographic images of holes side-walls in Ni-based superalloy. (**a**): 20,000 pulses (**b**): 50,000 pulses (**c**): 100,000 pulses (**d**): 200,000 pulses (**e**): 500,000 pulses.

For the holes in the Ni-based superalloy, when the laser fluence was 4.51 J/cm^2^, as shown in Figure 10, there were recast layers on the side-wall surfaces. These recast layers were mainly located in the lower part of the holes. In fact, the recast layer for the blind-hole ablation was more likely to be induced. The main reason is that the ablated materials could not be completely ejected through the cavity entrance, they re-solidified and then deposited on the side-wall surface as the recast layer. Furthermore, with the hole depth increasing, the blind hole entrance diameters increased, but the exit diameters became small quickly showing from 71 to 28 µm, resulting in the substantial taper as shown in Figure 10a–d. At last, when the pulses were 500,000, the through-hole was processed, the exit diameter began to increase, but the recast layer located in the lower part of the hole still existed as shown in Figure 10e.

**Figure 11:**
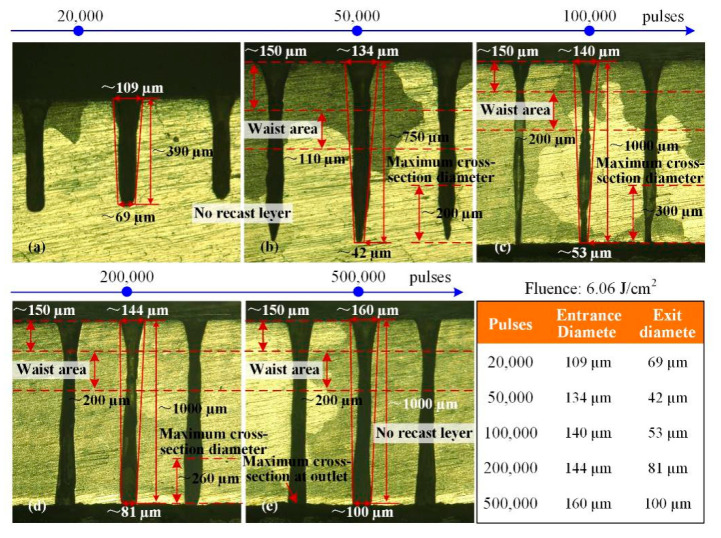
Metallographic images of holes side-walls in Ni-based superalloy. (**a**): 20,000 pulses (**b**): 50,000 pulses (**c**): 100,000 pulses (**d**): 200,000 pulses (**e**): 500,000 pulses.

For the higher laser fluence of 6.06 J/cm^2^, high quality blind-holes without the recast layer could be ablated as shown in Figure 11a,b. At the same time, the hole exit diameters also became small with the increase in hole depth. As the through-holes were created, there were recast layers in the lower parts of the through-hole as shown in Figure 11c. After that, with the increasing pulses, the through-holes entrance and exit diameters increased from 140 µm to 160 µm and from 53 µm to 100 µm, respectively, as shown in Figure 11c–e. Moreover, the recast layer was completely cleaned off by the subsequent pulses ablation as shown in Figure 11e. That is, the ablated materials could flow out and away from the hole exits completely. Overall, for the high laser fluence, a high-quality blind- or through-hole can be created without the recast layer, and the taper of holes also was improved. Similar to ablation of ceramic TBCs, the hole ablation in Ni-based superalloy had very high repeatability which is important for precision industrial applications.

Furthermore, for the holes shown in Figure 11, after 50,000 pulses, there were the waist areas, that is, the small cross-section diameter in the upper portion of the holes, and the phenomenon was not found for the holes shown in Figure 10. A similar phenomenon was found in the research of Leitz et al. [31] and Luft et al. [32], the lasers used were picosecond lasers and femtosecond lasers operating at 10 ps and 200 fs pulse duration, respectively. This phenomenon may be related to the existence of plasma and multiple reflections of the laser inside the hole [31]. It could be seen that the length of waist area and the distance from the material surface to the waist area were about 110 µm and 150 µm, respectively, for the blind-holes as shown in Figure 11b; the hole diameter of the lower part was large, the distance between the maximum cross-section diameter and the hole bottom was about 200 µm. Thereafter, as the pulses increased, the through-holes were ablated as shown in Figure 11c–e. The length of waist area increased to 200 µm, but the distances from the material surface to the waist area were still about 150 µm, although the cross-section diameters of the holes varied greatly. The holes diameters of the lower part also were large, and the distances between the maximum cross-section diameter and hole bottom are from 300 µm to 260 µm, then to zero, that is, the maximum cross-section diameter was the exit diameter.

**Figure 12:**
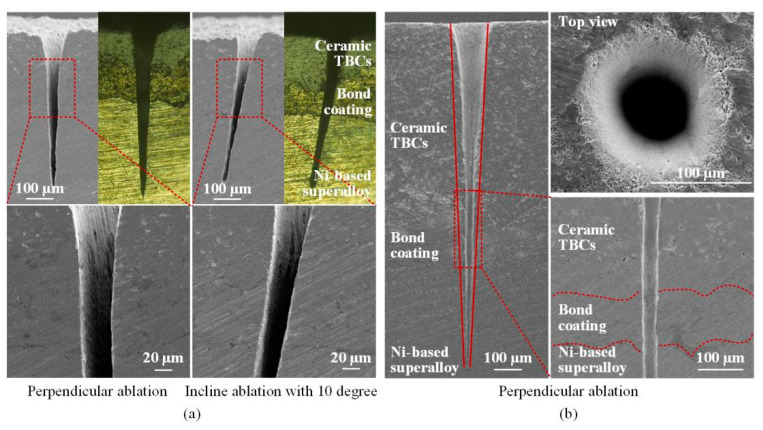
SEM and metallographic images of microstructures in Ni-based superalloy coated with ceramic TBCs. (**a**): Groove ablation (**b**): Hole drilling.

Finally, high-quality hole and groove in Ni-based superalloy coated with ceramic TBCs could be created using picosecond ultrashort pulse laser, as shown in Figure 12. The processing parameters were a wavelength of 1064 nm, a fluence of 6.06 J/cm^2^, a scanning speed 0.1 mm/s and 100,000 pulses for the Figure 12a,b, respectively. It was found that there were no recast layers on the grooves side-wall surfaces, no delamination and micro-cracks at the combined positions between the multilayer materials for both the perpendicular and incline ablations. For the drilled holes the entrance surfaces were clean and free of molten spatters, and the side-wall morphology was smooth, delicate and free of delamination and micro-cracks. In addition, the hole in Figure 12b was conical and was similar to those in Figure 10, even though the hole material in Figure 12b was a Ni-based superalloy with TBCs, while in Figure 10 it was metal Ni. In the Ni-based superalloy laser processing, the hole was conical before the through-hole formed, as shown in Figure 10c. With the laser processing parameters increased, the through-hole formed and the diameter of the hole bottom gradually increased, and the roundness of the hole became better, as shown in Figure 11e. As for Figure 12, the holes resembled the ones from Figure 10, as the laser processing parameters increasing, the side-wall of the through-hole will may be similar to that of the hole in Figure 11. In the follow-up work, the laser processing parameters will continue to increase, and holes similar to Figure 11, showing the high cylindricity, which would be researched in the follow-up work.

Overall, there are no micro-cracks and interfacial cracks on the hole side-wall surface in Ni-based superalloy with ceramic TBCs ablated by using ultrashort pulse laser, no matter whether in this article or other scholars’ papers, such as Das et al. (the laser pulse duration of 150 fs) [20], Feng et al. (the laser pulse duration of 150 fs) [21] and Sun et al. (the laser pulse duration of 10 ps) [33]. However, such defects have been found when using longer pulse duration laser processing. For example, during millisecond pulse laser processing, there are a large number of micro-cracks [34,35], and the interfacial cracks size are about 3 to 12 μm [34]. It can be found that the number of cracks on the hole side-wall surface are significantly reduced during nanosecond pulse laser processing [36]. Combined with ultrashort pulse laser processing, there are fewer or even no cracks on the hole side-wall surface as the pulse duration becoming shorter, indicating the advantages of ultrashort pulse laser.

## 4. Conclusions

For the potential applications to blade film cooling holes, percussion drilling is presented using picosecond ultrashort pulse laser in Ni-based superalloy, ceramic TBCs and ceramic TBCs/substrate multilayer material. First, the effects of threshold and wavelength on hole diameter have been discussed. The results show that the laser with 1064 nm wavelength need be employed to drill the holes with the diameter larger than 100 µm. Then, deep hole ablation with 1064 nm wavelength has been performed. The hole dimension and morphological characteristics have been analyzed for multiple processing parameters. It could be found that high processing efficiency could be expected for the high laser fluence. For example, the processing time for a through-hole in Ni-based superalloy with a thickness of 1 mm is 500 s for a laser fluence of 4.51 J/cm^2^, and is only 100 s for a fluence of 6.06 J/cm^2^. Processing efficiency of through-hole in ceramic TBCs with a thickness of 0.7 mm has been increased by 4 times for a fluence of 6.06 J/cm^2^ than 4.51 J/cm^2^. The holes morphologies are illustrated in two materials, including the holes surfaces and the side-wall morphologies. The results also show, for the higher laser fluence, the blind- and through-hole could be processed without recast layer and the taper also has been improved. Combined with the high ablation efficiency for the high laser fluence, it can be concluded that high-quality and efficient processing can be achieved with high fluence using picosecond ultrashort pulse laser. Finally, a high quality hole in Ni-based superalloy coated with ceramic TBCs was processed without molten spatter around the periphery of the hole entrance and recast layer on the side-wall surface.

## References

[1] Ghosh S. (2015). Thermal Barrier Ceramic Coatings—A Review. Advanced Ceramic Processing.

[2] Goswami B., Ray A., Sahay S. (2004). Thermal Barrier Coating System for Gas Turbine Application–A Review. High Temp. Mater. Process..

[3] Tang F., Ajdelsztajn L., Kim G.E., Provenzano V., Schoenung J.M. (2006). Effects of variations in coating materials and process conditions on the thermal cycle properties of NiCrAlY/YSZ thermal barrier coatings. Mater. Sci. Eng. A.

[4] Meier S.M., Gupta D.K., Sheffler K.D. (1991). Ceramic thermal barrier coatings for commercial gas turbine engines. JOM.

[5] Teng S., Han J.-C., Poinsatte P.E. (2001). Effect of Film-Hole Shape on Turbine-Blade Film-Cooling Performance. J. Thermophys. Heat Transf..

[6] Beck T. (2011). Laser drilling in gas turbine blades. Laser Tech. J..

[7] Girardot J., Schneider M., Berthe L., Favier V. (2013). Investigation of delamination mechanisms during a laser drilling on a cobalt-base superalloy. J. Mater. Process. Technol..

[8] Sun X., Wang W., Mei X., Pan A., Liu B., Li M. (2019). Controllable dot-matrix marking on titanium alloy with anti-reflective micro-structures using defocused femtosecond laser. Opt. Laser Technol..

[9] Corcoran A., Sexton L., Seaman B., Ryan P., Byrne G. (2002). The laser drilling of multi-layer aerospace material systems. J. Mater. Process. Technol..

[10] Zhao W., Wang L. (2018). Microdrilling of Through-Holes in Flexible Printed Circuits Using Picosecond Ultrashort Pulse Laser. Polym..

[11] Gautam G.D., Pandey A.K. (2018). Pulsed Nd:YAG laser beam drilling: A review. Opt. Laser Technol..

[12] Dausinger F., Hügel H., Konov V.I. (2003). Micromachining with ultrashort laser pulses: From basic understanding to technical applications. SPIE Proc..

[13] Sun R., Zhang X., Cao W., Gong S., Zhang X. (2013). Characteristic of Hole Wall Trepanning by Picosecond Laser in Superalloy. Rare Metal Mat. Eng..

[14] Sun R., Zhang X., Cao W., Gong S. (2014). Laser Drilling of Ni-Base Single-Crystal Superalloy through Thermal Barrier Coatings. Rare Metal Mat. Eng..

[15] Wang R., Duan W., Wang K., Dong X., Fan Z., Mei X., Wang W., Zhang S. (2018). Computational and experimental study on hole evolution and delamination in laser drilling of thermal barrier coated nickel superalloy. Opt. Lasers Eng..

[16] Qi H., Lai H. (2012). Micromachining of Metals and Thermal Barrier Coatings using a 532nm Nanosecond Fiber Laser. Phys. Procedia.

[17] Fan Z., Duan W., Zhang X., Mei X., Wang W., Cui J. (2019). Influence of Preheating on the Microstructure Evolution of Laser Re-Melting Thermal Barrier Coatings/Ni-Based Single Crystal Superalloy Multilayer System. Materials.

[18] Zhang J., Long Y., Liao S., Lin H.-T., Wang C. (2017). Effect of laser scanning speed on geometrical features of Nd:YAG laser machined holes in thin silicon nitride substrate. Ceram. Int..

[19] Wang M., Yang L., Zhang S., Wang Y. (2018). Experimental investigation on the spiral trepanning of K24 superalloy with femtosecond laser. Opt. Laser Technol..

[20] Das D.K., Pollock T.M. (2009). Femtosecond laser machining of cooling holes in thermal barrier coated CMSX4 superalloy. J. Mater. Process. Technol..

[21] Feng Q., Picard Y.N., McDonald J., Van Rompay P., Yalisove S., Pollock T. (2006). Femtosecond laser machining of single-crystal superalloys through thermal barrier coatings. Mater. Sci. Eng. A.

[22] Zhao W., Wang W., Jiang G., Li B.Q., Mei X. (2015). Ablation and morphological evolution of micro-holes in stainless steel with picosecond laser pulses. Int. J. Adv. Manuf. Technol..

[23] Semaltianos N., Perrie W., French P., Sharp M., Dearden G., Logothetidis S., Watkins K.G. (2008). Femtosecond laser ablation characteristics of nickel-based superalloy C263. Appl. Phys. A.

[24] Tan B., Dalili A., Venkatakrishnan K. (2008). High repetition rate femtosecond laser nano-machining of thin films. Appl. Phys. A.

[25] Sivayoganathan M., Tan B., Venkatakrishnan K. (2012). Effect of mega-hertz repetition rate on the agglomerated particle size of femtosecond synthesized nanostructures. Opt. Mater. Express.

[26] Zhao W., Yu Z. (2018). Self-cleaning effect in high quality percussion ablating of cooling hole by picosecond ultra-short pulse laser. Opt. Lasers Eng..

[27] Chien W.-T., Hou S.-C. (2006). Investigating the recast layer formed during the laser trepan drilling of Inconel 718 using the Taguchi method. Int. J. Adv. Manuf. Technol..

[28] Li C., Wang W. (2004). Quantitative characterization of lamellar microstructure of plasma-sprayed ceramic coatings through visualization of void distribution. Mater Sci. Eng. A.

[29] Wang X., Lim G., Zheng H., Ng F., Liu W., Chua S. (2004). Femtosecond pulse laser ablation of sapphire in ambient air. Appl. Surf. Sci..

[30] Garofano J.K., Marcus H.L., Aindow M. (2009). Nanoscale carbide precipitation in the recast layer of a percussion laser-drilled superalloy. Scr. Mater..

[31] Leitz K.-H., Redlingshöfer B., Reg Y., Otto A., Schmidt M. (2011). Metal Ablation with Short and Ultrashort Laser Pulses. Phys. Procedia.

[32] Luft A., Franz U.A. (1996). Emsermann, J. Kaspar, A study of thermal and mechanical effects on materials induced by pulsed laser drilling. Appl. Phys. A.

[33] Sun X., Dong X., Wang K., Wang R., Fan Z., Duan W. (2019). Experimental investigation on thermal effects in picosecond laser drilling of thermal barrier coated In718. Opt. Laser Technol..

[34] Wang R., Wang K., Dong X., Fan Z., Duan W., Me X., Wang W., Cui J., Zhang S. (2018). An experimental investigation into the defects of laser-drilled holesin thermal barrier coated Inconel 718 superalloys. Int. J. Adv. Manuf. Tech..

[35] Fan Z., Dong X., Wang K., Duan W., Wang R., Mei X., Wang W., Cui J., Yuan X., Xu C. (2016). Effect of drilling allowance on TBC delamination, spatter and re-melted cracks characteristics in laser drilling of TBC coated superalloys. Int. J. Mach. Tools Manuf..

[36] Lv J., Dong X., Wang K., Duan W., Fan Z., Mei X. (2016). Study on process and mechanism of laser drilling in water and air. Int. J. Adv. Manuf. Technol..

